# Resonant Doping in Binary Sb(V)-Oxide Sb_2_O_5_ for High-Mobility Transparent Conductors

**DOI:** 10.1021/acs.chemmater.6c01056

**Published:** 2026-09-11

**Authors:** Ke Li, Romain Claes, Alexander G. Squires, David O. Scanlon

**Affiliations:** † Department of Chemistry, 4919 University College London, 20 Gordon Street, London WC1H 0AJ, UK; ‡ School of Chemistry, University of Birmingham, Edgbaston, Birmingham B15 2TT, UK

## Abstract

The development of earth-abundant transparent conducting oxides (TCOs) requires materials that combine high carrier concentrations with minimal degradation of electron mobility. Sb­(V)-based oxides have recently emerged as promising candidates owing to their highly dispersive Sb 5*s*-derived conduction bands and deep valence-band positions. While degenerate conductivity in Sb_2_O_5_ has previously been predicted through anion-site F substitution, the viability of resonant donor engineering on the cation sublattice remains unexplored. Here, using hybrid density functional theory and rigorous defect thermodynamics, we show that W acts as an effective resonant donor dopant when substituted on Sb sites in Sb_2_O_5_, whereas Mo exhibits only weak donor behavior due to the stabilization of the localized electronic state for the neutral charge state. Both W_Sb_ and Mo_Sb_ exhibit low formation energies under n-type conditions, with W-doped Sb_2_O_5_ yielding an electron concentration exceeding 1 × 10^19^ cm^–3^ while Mo-doping results in a moderate value of around 1 × 10^18^ cm^–3^. Unfolded band structure analysis reveals that the W 5*d* and Mo 4*d* donor states reside well above the conduction band minimum and exhibit negligible hybridization with the Sb 5*s* states that govern transport. This preservation of the dispersive conduction band enables degenerate doping without significant perturbation of band curvature. These results establish resonant cation-site doping as a transferable design strategy in Sb­(V)-based oxides and broaden the chemical space for high-mobility, earth-abundant TCOs, while recognizing monoclinic Sb_2_O_5_ as an experimentally realized but synthetically challenging host material.

## Graphical abstract

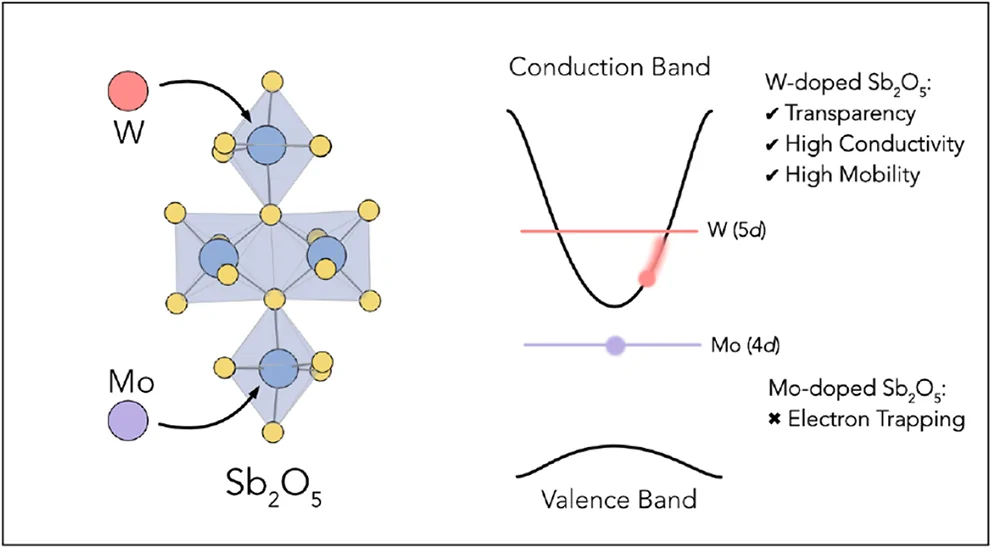

## Introduction

Transparent conducting oxides (TCOs) bridge the gap between optical transparency and high electrical conductivity, underpinning various optoelectronic devices such as solar cells, low-emissivity windows, and touchscreens.^1–5^ TCOs are typically categorized into *n*-type and *p*-type, with *n*-type materials being far more prevalent and easier to design due to the cation *s*-orbital-dominated conduction bands being highly dispersive, which can host delocalized electrons.^1,6–8^ Whereas the O 2*p* valence band is relatively localized, making the high-mobility *p*-type TCOs considerably more challenging.^9–12^ Sn-doped In_2_O_3_ (ITO), F-doped SnO_2_ (FTO) and Sb-doped SnO_2_ (ATO) are well-known conventional *n*-type TCOs with superior conductivities,^1,13–15^ while limitations on mobilities were reported for high carrier concentrations due to self-compensation and perturbation to the host cation *s*-states at the conduction band edge.^16–19^

To overcome the limitations of conventional dopants, a resonant doping strategy has been explored in several well-established TCO systems. Transition-metal dopants introduce donor *d*-states located well above the conduction band minimum (CBM), interacting only weakly with the host cation *s* states that dominate the conduction band edge. Mo-doped In_2_O_3_ was reported to exhibit mobility nearly twice that of conventional ITO,^16^ and W-doped SnO_2_ similarly exhibited enhanced carrier mobility relative to conventional F-doped SnO_2_, associated with reduced ionized impurity scattering.^20^ Comparable behavior has also been observed in Ta-doped SnO_2_, Zr- and Ce-doped In_2_O_3_ and La-doped BaSnO_3_, where all preserve high mobility while achieving high carrier concentrations sufficient for degenerate *n*-type conductivity.^17,21–26^

Recent experimental and computational studies have identified Sb­(V)-based oxides as potential earth-abundant TCOs.^27–31^ Their conduction bands are predominantly derived from the Sb 5*s* orbitals, forming a highly dispersive band edge. Our previous investigation of the under-explored binary system Sb_2_O_5_ revealed a wide *n*-type doping window of around 5 eV and demonstrated that conventional anion-site F doping can raise the electron concentration sufficiently to produce degenerate conductivity. Its favorable electronic structure and wide *n*-type doping window motivate the computational exploration of alternative donor design strategies in Sb­(V)-based oxides.

Building upon our previous study and motivated by the success of resonant doping in conventional TCOs, we investigate Mo and W as cation-site transition-metal donors on the Sb^5+^ site in Sb_2_O_5_ using hybrid density functional theory. Despite their similar low formation energies and nominal donor characters, Mo and W exhibit qualitatively different electronic behavior. Mo_Sb_ is stabilized in its neutral charge state, forming a localized dopant-derived state within the band gap that traps the donated electron, whereas W_Sb_ remains ionized and effectively donates electrons into the conduction band. The unfolded band structure shows that the W-derived *d*-states lie well above the CBM and show negligible hybridization with the conduction band edge. Under favorable *n*-type growth conditions, W doping produces electron concentrations exceeding 10^19^ cm^–3^, above the predicted metal-insulator transition threshold, while retaining an electronic structure favorable for preserving the dispersive host conduction band edge. These findings establish a cation-site resonant doping mechanism that is fundamentally distinct from the conventional F anion doping investigated previously, and provide a potentially transferable design principle for high-mobility, earth-abundant Sb­(V)-based transparent conducting oxides.

## Computational Methodology

All DFT calculations were performed using the Vienna *ab initio* Simulation Package (VASP) with the PBE0 hybrid functional, consistent with our previous studies.^32–36^ Defect calculations were done using the supercell approach, where the same 112-atom bulk supercell was employed as in the previous F-doped Sb_2_O_5_ study.^27^ For each defect, 10 distorted structures were generated using the ShakeNBreak approach and initially relaxed using Γ-point only sampling to identify the ground-state configuration.^37,38^ Structural relaxations with an energy cutoff of 500 eV and a Γ-centered 2 × 2 × 2 *k*-point mesh were performed to obtain the total energy of the ground-state defective supercell. Spin-orbit coupling (SOC) was not included in these defect calculations, in line with previous defect studies of related Sb­(V)-based oxides.^27–30^ SOC is not expected to qualitatively alter the defect thermodynamics regarding the doping behavior discussed here. The formation energy of a defective system can then be calculated using the following equation.^39^

1 $$\Delta E_{f}^{\left(X,q\right)}=E^{\left(X,q\right)}-E^{bulk}+\underset{i}{\sum }n_{i}\mu_{i}+qE_{F}+E^{corr}$$

The first two terms describe the energy difference between the defective supercell with charge state *q* and the bulk supercell. The third term accounts for the change in energy when removing or adding an atom with the external reservoirs, where *μ*_*i*_ represents the corresponding atomic chemical potential. *qE*_*F*_ describes the Fermi energy accounting for the electrons. An additional correction term is applied to compensate for spurious electrostatic interactions arising from the finite supercell size. The chemical potential limits used in the defect formation energy calculations were determined from the thermodynamic stability region of Sb_2_O_5_. This was obtained by comparing the calculated formation energy of Sb_2_O_5_ against all relevant competing phases in the Sb–O chemical space. For W- and Mo-doped Sb_2_O_5_, W- and Mo-containing secondary phases were also included. The Python package doped was used for pre- and post-processing.^40^ Additional details of the competing phases’ formation energies, chemical potential limits and limiting phases are provided in Tables S1, S2, S4 and S5.

To relate the calculated chemical potential limits to experimentally relevant synthesis conditions, the oxygen partial pressure corresponding to the calculated oxygen chemical potential was estimated using the Shomate equation as implemented in the Spinney package:^41^

2 $$\mu \left(T,\;p\right)=h\left(0,\;p^{{}^{\circ}}\right)+\left[g\left(T,\;p^{{}^{\circ}}\right)-g\left(0,\;p^{{}^{\circ}}\right)\right]+k_{B}T\ln\left(\frac{p}{p^{{}^{\circ}}}\right)$$

where *p*° is the standard pressure, *g* is the molar Gibbs free energy and *h*(0, p°) is the molar enthalpy at 0 K and standard pressure. For the calculated oxygen chemical potential (*μ*_*O*_ = −0.76 eV), the corresponding oxygen partial pressures between 600 and 700 °C were estimated to range from 1.22 × 10^2^ to 1.45 × 10^3^ atm.

The equilibrium defect concentrations were evaluated within the dilute defect approximation using Boltzmann statistics according to^42^

3 $$\left[X^{q}\right]=N_{X^{q}}g\exp\left(\frac{-\Delta E_{f}^{\left(X,\;q\right)}}{k_{B}T}\right)$$

where $N_{X^{q}}$ is the concentration of available sites for defect species *X*, *g* is the defect degeneracy, and $E_{f}^{\left(X,\;q\right)}$ is the defect formation energy. To estimate room-temperature defect and carrier concentrations, the “frozen defect approach” was employed, in which the total concentration of each defect was fixed to its equilibrium concentration at the annealing temperature while the relative populations of the different charge states and the carrier concentrations were allowed to re-equilibrate during the cooling process to room temperature (300 K).^27,40,43^ The unfolded band structures were calculated and plotted using easyunfold.^44^ Defect calculations were carried out in line with the recently established defect simulation guidelines to ensure reproducibility, with all relevant metadata and calculation data available on Zenodo (10.5281/zenodo.19234120).^39^

## Doping Behavior of W and Mo in Sb_2_O_5_

In our recent work, we investigated the under-explored binary Sb­(V)-based oxide Sb_2_O_5_ using hybrid DFT calculations.^27^ Sb_2_O_5_ crystallizes in a monoclinic *C*2/*c* structure and exhibits a wide effective optical band gap of around 3.6 eV, which allows for transparency.^27^ Similar to other post-transition metal *n*-type TCOs, we observed a dispersive CBM dominated by the Sb 5*s* states, yielding a low average effective mass of 0.31 *m*_*e*_.^27^ F-doping on the oxygen site formed low-energy substitution defects that pushed the self-consistent Fermi level into the conduction band, achieving degenerate conductivity with electron concentrations exceeding 10^19^ cm^–3^.^27^ Building on these findings, we therefore explored cation-site transition-metal donors as a distinct doping strategy for Sb_2_O_5_, to determine whether the dopants act as resonant donors or instead form reduced dopant centers by forming localized in-gap states.

All intrinsic point defects considered here are taken from our previous study.^27^ For consistency with our other Sb­(V)-based oxide studies, a +1 charge state was added to *V*_Sb_ and −1 charge states for oxygen vacancies, following the charge state guessing feature in doped.^40^ As shown in Figure 1, only one acceptor defect is present in Sb_2_O_5_, namely *V*_Sb_. A wide *n*-type doping window of ∼5 eV is observed by the intersection of *V*_Sb_ with the CBM, indicating excellent potential for extrinsic doping. To substitute the high-valent Sb^5+^ cation, we selected W and Mo as candidate transition-metal dopants that can access oxidation states compatible with substitution on the Sb^5+^ site. The W- and Mo-doped Sb_2_O_5_ transition level diagrams under the metal-rich/O-poor conditions are shown in Figure 1a and b, respectively. The selected chemical potential limit corresponds to the most *n*-type growth condition within the thermodynamic stability region of Sb_2_O_5_. As discussed in the Computational Methodology section, the calculated oxygen chemical potential of −0.76 eV corresponds to estimated oxygen partial pressures of 1.22 × 10^2^ to 1.45 × 10^3^ atm over 600 °C to 700 °C.^46–49^ These values indicate strongly oxidizing conditions and are consistent with previous high-pressure synthesis studies reporting that stabilization of monoclinic Sb_2_O_5_ requires approximately 200 MPa oxygen pressure (1.97 × 10^3^ atm) at 873 K.^46,47^ It is important to note that these estimated oxygen pressures correspond to equilibrium thermodynamic conditions and should not be interpreted as a unique experimental processing requirement, as non-equilibrium synthesis may access metastable phases or oxidation states under conditions distinct from those predicted by equilibrium thermodynamics. Nevertheless, monoclinic Sb_2_O_5_ should presently be regarded as an experimentally realized but synthetically challenging TCO host material, for which no scalable synthesis route has yet been demonstrated. High-pressure synthesis or oxidation-based routes followed by annealing may therefore provide possible directions for future experimental validation.^46,47,50,51^ In Figure 1, both W_Sb_ and Mo_Sb_ substitution defects exhibit the lowest formation energies among all the defects at their respective self-consistent Fermi levels, which were determined at an annealing temperature of 600 °C to 700 °C, consistent with our previous study, which was based on experimental annealing conditions.^27,43,46,48,49^ Both substitution defects are thermodynamically stable and are not charge compensated by the acceptor defects under these conditions. However, the (+1/0) transition levels of W_Sb_ and Mo_Sb_ show a clear distinction, with W_Sb_ located at 0.51 eV above the CBM, while Mo_Sb_ is pinned at the band edge.

**Figure 1:**
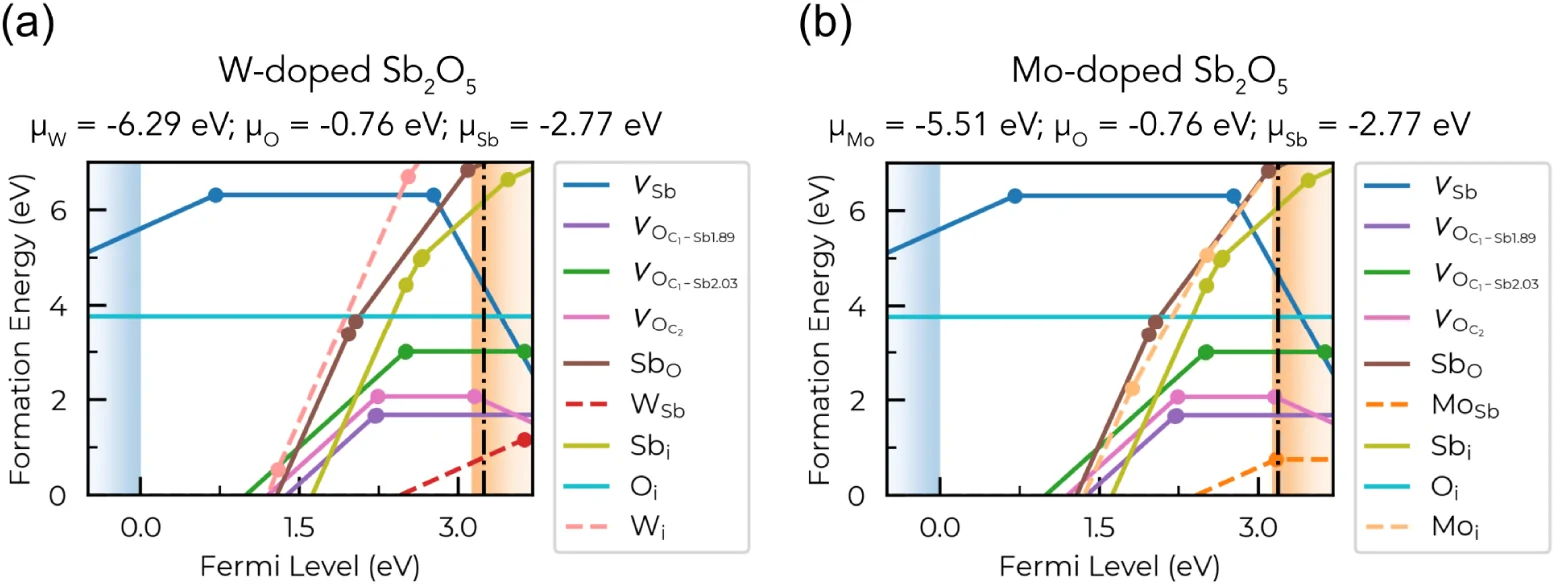
Transition level diagrams for (a) W-doped and (b) Mo-doped Sb_2_O_5_ under Sb-rich/O-poor conditions (the most *n*-type condition). The self-consistent Fermi levels for W- and Mo-doping were predicted to lie at 0.13 and 0.06 eV above the CBM, shown in black dotted lines.

To understand the origin of the less effective donor behavior for Mo_Sb_, the eigenvalue-based electronic structure analysis with charge density plots is shown in Figure 2. The result reveals the presence of an occupied defect level located deep within the band gap, around 1.08 eV below the CBM. As shown in Table 1, this state is dominated by Mo 4*d* contributions, with a minor degree of mixing from neighboring O *p* orbitals. The charge density analysis of this localized state is illustrated in Figure 2b, which indicates strong localization around the Mo center and its coordinating oxygen atoms. Such localization stabilizes the neutral charge state of Mo_Sb_ through electron trapping on the Mo site, corresponding to reduction of the Mo dopant. A direct comparison with W_Sb_, shown in Figure 3, further clarifies the origin of the contrasting donor behavior. The projected density of states (Figure 3a) shows an in-gap Mo-derived *d* state, whereas no equivalent localized W *d* state is observed, indicating a greater tendency for electron trapping on Mo. The COHP analysis in Figure 3b shows that this Mo *d* state is associated with antibonding-like Mo–O *d*-*p* interactions around the substitution site. This is further supported by the averaged bonding parameters in Table 2, where reduction from Mo^6+^ to Mo^5+^ is accompanied by Mo–O bond elongation, less negative ICOHP and a lower ICOBI, while the corresponding W–O parameters remain almost unchanged. These results show that Mo stabilizes the reduced 5+ state through electron trapping, whereas W does not form an analogous localized reduced state and instead donates electrons more effectively to the conduction band. Despite the similar nominal donor character of W and Mo, this contrast demonstrates that donor effectiveness is governed by the stability of the reduced dopant state and its local bonding environment. This distinction is also reflected in the predicted self-consistent Fermi level, which lies deeper in the conduction band for W-doped Sb_2_O_5_ than for Mo-doped Sb_2_O_5_. The corresponding W and Mo interstitials are also donor-like defects, while their high formation energies preclude any significant contribution to the electron concentration.

**Table 1:** Summary of Band-Edge Character and Localized Defect States for Neutral Mo_Sb_ in Mo-Doped Sb_2_O_5_, Obtained from Spin-Polarized Eigenvalue Analysis Generated by doped^40,45^ ^a^

State	Energy (eV)	P-ratio	Dominant orbital character
VBM	3.97	0.04	O *p* (0.75)
CBM	7.21	0.04	Sb *s* (0.38), O *s* (0.20)
Localized orbitals	6.12	0.88	Mo *d* (0.56), O *p* (0.26)

a Energies are referenced to the VBM

**Table 2:** Summary of the Average Dopant-Oxygen Bonding Interactions for W_Sb_ and Mo_Sb_ in Sb_2_O_5_, Obtained from COHP Analysis^a^

Oxidation state	Bond	ICOHP (eV)	ICOBI	Bond length (Å)
5+	W–O	–5.347	0.745	1.941
6+	W–O	–5.352	0.746	1.943
5+	Mo–O	–4.562	0.631	2.002
6+	Mo–O	–5.440	0.778	1.942

a ICOHP values, ICOBI values and bond lengths are averaged over the six nearest-neighbor dopant-O bonds.

**Figure 2:**
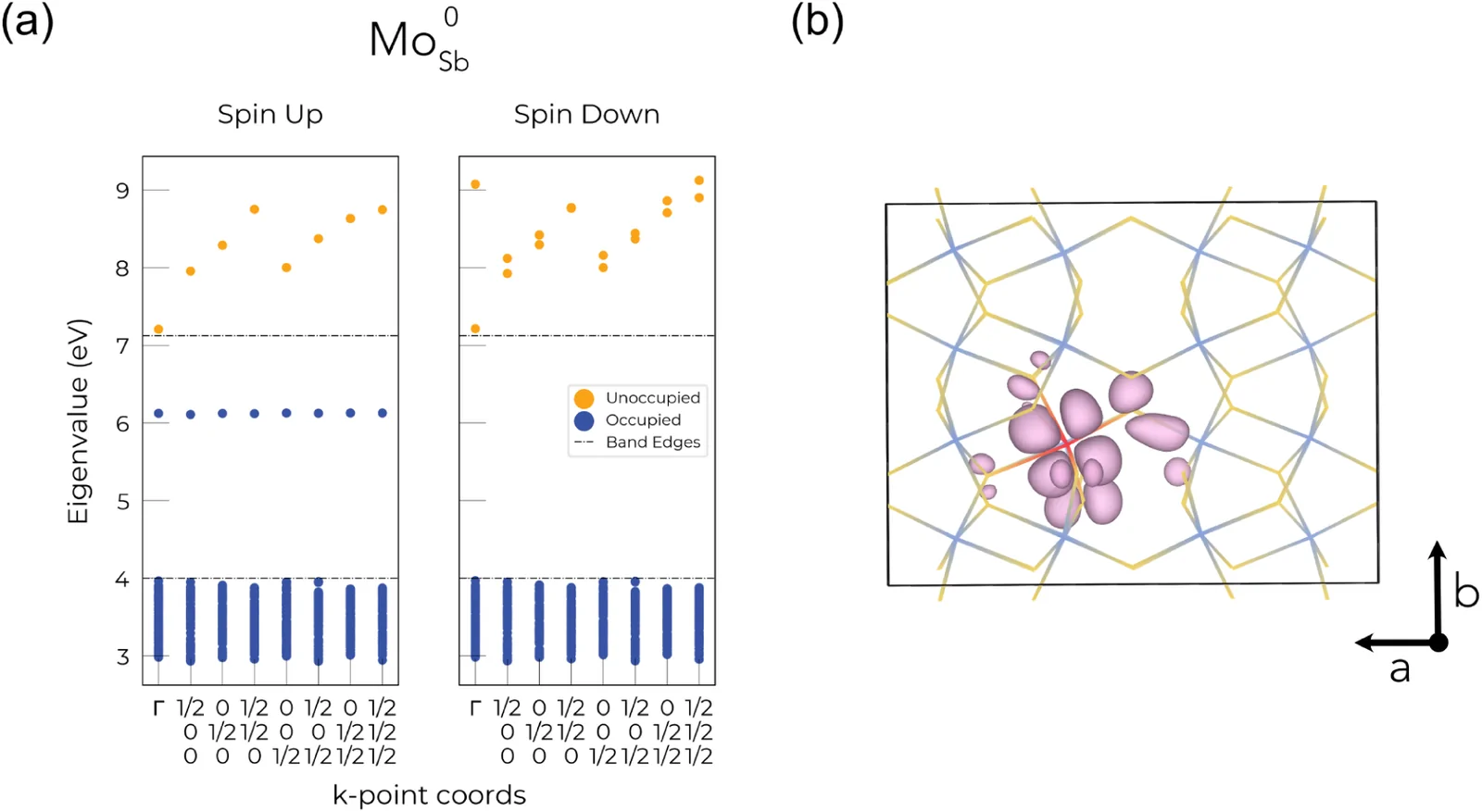
(a) Spin-polarized eigenvalues of the neutral Mo_Sb_ defect, plotted using doped.^40,45^ Occupied states are shown in blue and unoccupied states in orange. The dashed lines indicate the VBM and CBM. (b) Charge density isosurface showing the localized state of Mo_Sb_^0^, displayed using a wireframe structure with an isosurface level of 0.015 Å^–3^. The blue, yellow and red lines represent the Sb, O and Mo atoms, respectively.

**Figure 3:**
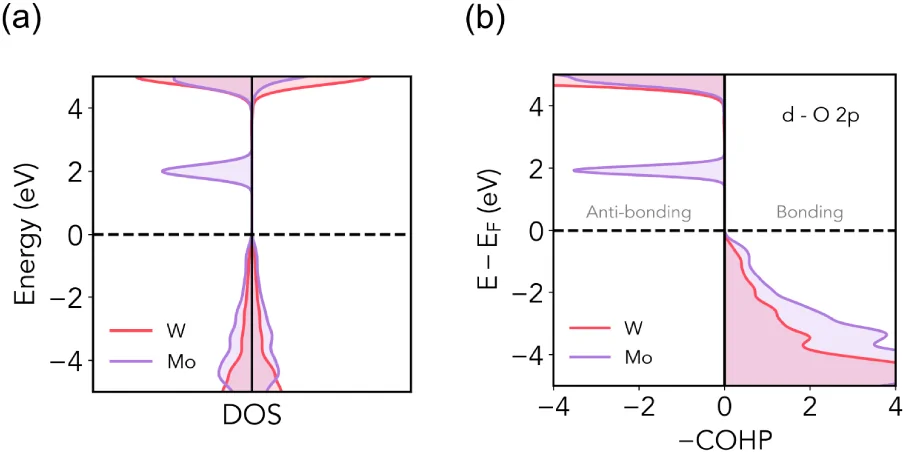
(a) Comparison of the projected density of states of W- and Mo-doped Sb_2_O_5_, showing the relative energetic positions of the W- and Mo-derived *d*-states. (b) Crystal orbital Hamilton population (COHP) analysis of the interactions between the dopant *d* states and the O 2*p* states of the six neighboring oxygen atoms for Mo_Sb_ and W_Sb_.

Figure 4a and b show the temperature-dependent defect concentrations together with the corresponding quenched and annealed electron concentrations. Annealing/growth temperatures between 600 °C and 700 °C were considered based on experimentally reported values for Sb_2_O_5_.^27,46–49^ Figure 4a shows that W_Sb_ dominates among all defects under the most *n*-type conditions, with its concentration increasing significantly with annealing temperatures. The shaded blue region corresponds to the experimentally reported annealing temperature range,^27,46–49^ where the W_Sb_ concentration is comparable to the resulting electron concentration, reaching 1 × 10^19^ cm^–3^. Notably, the electron concentrations during annealing and upon cooling remain essentially unchanged and therefore overlap in Figure 4a. This behavior reflects that W_Sb_ is stabilized in its +1 charge state following charge-state re-equilibration at 300 K. In Figure 4b, the quenched electron concentration in Mo-doped Sb_2_O_5_ is consistently lower than the corresponding annealed concentration, despite the increasing concentration of Mo_Sb_ with annealing temperature. This reduction in the quenched electron concentration is consistent with the (+1/0) transition level of Mo_Sb_ lying at the conduction band edge and with the localized Mo-derived in-gap state identified above, which together favor electron trapping upon cooling. Nevertheless, an electron concentration of over 1 × 10^18^ cm^–3^ is still attainable within the typical annealing temperature range for Mo-doped Sb_2_O_5_. Therefore, W-doped Sb_2_O_5_ reaches the degenerate *n*-type regime, with an electron concentration exceeding 10^19^ cm^–3^, approximately one order of magnitude above the Mott criterion value of 1.08 × 10^18^ cm^–3^ estimated in our previous study.^27^ In contrast, Mo doping yields substantially lower free-carrier concentrations because of dopant-centered electron trapping.

**Figure 4:**
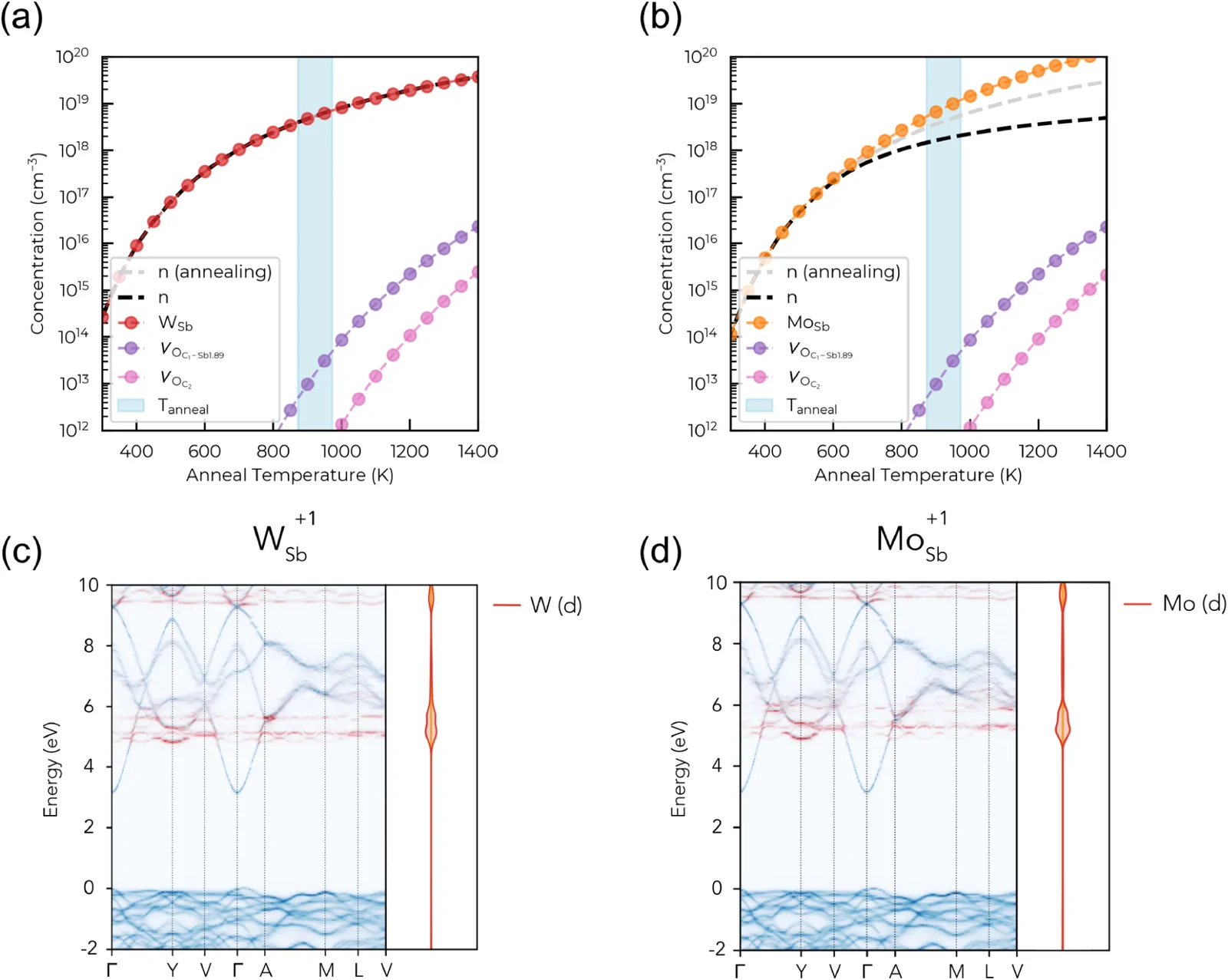
(a, b) Temperature-dependent defect and electron concentrations in (a) W-doped and (b) Mo-doped Sb_2_O_5_ under the *n*-type growth conditions. The dashed gray lines show the equilibrium electron concentration during annealing. The black dashed lines denote the room-temperature electron concentration obtained using the frozen-defect (quenched) approach. The blue shaded region indicates the typical experimental annealing temperature range of 600 °C to 700 °C.^27,46–49^ Defects with concentrations less than 10^12^ cm^–3^ are omitted from the legend. (c, d) Unfolded band structures with dopant *d*-orbital projected density of states for (c) W_Sb_^+1^ and (d) Mo_Sb_^+1^ in Sb_2_O_5_. The contributions from W and Mo *d* orbitals are highlighted in red.

To investigate the effects of W and Mo on the band structure of Sb_2_O_5_, the unfolded band structures and projected density of states for the substitution defects (W_Sb_ and Mo_Sb_) in the fully ionized (+1) charge states are shown in Figure 4c and d. In both cases, the overall band dispersion remains largely unchanged compared to the pristine host, with the dopant-derived *d* states located approximately 2 eV above the CBM and showing negligible hybridization with the highly dispersive Sb 5*s*-dominated conduction band edge. This behavior is characteristic of resonant dopants and is favorable for preserving the intrinsic high mobility upon doping. For W_Sb_, this resonant doping behavior is consistent with its thermodynamic stability in the fully ionized state and its effective donation of electrons to the conduction band. In contrast, although the ionized Mo_Sb_ exhibits a similar high-lying *d*-state configuration, Mo can undergo reduction to 5+ and form the localized in-gap state discussed above, thereby limiting its effectiveness as a donor defect.

The W-doping mechanism is therefore fundamentally distinct from the conventional anion-site F doping reported previously for Sb_2_O_5_.^27^ In the F-doped system, F substitutes on the O sites and contributes negligibly near the conduction band edge. In contrast, W substitutes directly on the Sb site while its localized *d* states remain energetically separated from the Sb 5*s*-derived CBM. The distinct electronic contributions of F, W, and Mo dopants are further illustrated by the comparative projected density of states shown in Figure S1. Such negligible interaction between W-derived *d*-states and the CBM reduces the perturbation of the host conduction band edge and is favorable for retaining its dispersive character. Although the predicted W-doped electron concentration is moderate compared with conventional high carrier concentration TCOs, it remains approximately one order of magnitude above the predicted metal-insulator transition threshold.^27^ Moreover, our previous transport calculations for Sb_2_O_5_ showed that ionized impurity scattering becomes the dominant mobility-limiting mechanism at carrier concentrations above 10^20^ cm^–3^.^27^ Operating within a more moderate carrier concentration regime may therefore help avoid the stronger ionized impurity scattering penalty associated with substantially higher concentrations of charged dopants. A mobility-led TCO design operating at moderate carrier concentrations may additionally reduce free-carrier absorption in the near-infrared compared with strategies that rely primarily on very high electron concentrations, which may be advantageous for applications such as photovoltaic transparent contacts.^52–54^ Indeed, for near-infrared transparent front contacts in tandem photovoltaics, moderate carrier concentrations combined with high mobility are actively preferred over ITO-like carrier densities, since sheet-resistance targets can be met while suppressing free-carrier absorption.^55,56^ This design principle is exemplified experimentally by high-mobility Zr- and Ce-doped In_2_O_3_ transparent electrodes used in silicon heterojunction and tandem photovoltaic applications.^23,57,58^ As a secondary practical consideration, cation-site doping using oxide-based W precursors may also avoid some of the processing challenges associated with volatile or corrosive fluorine-containing precursors and may enable more direct compositional control.^59–62^ Further experimental studies will be required to determine whether these potential processing advantages can be realized under the strongly oxidizing conditions required to stabilize monoclinic Sb_2_O_5_. Within these constraints, the present results demonstrate the versatility of Sb_2_O_5_ as a transparent conducting oxide host material, where both conventional anion-site doping and cation-site resonant doping can be employed to achieve high electron concentrations while preserving the favorable transport characteristics of the Sb 5*s*-derived conduction band.

## Conclusion

In summary, hybrid DFT calculations reveal that W acts as an effective resonant dopant when substituting Sb in Sb_2_O_5_, exhibiting low formation energies under *n*-type conditions. In contrast, Mo_Sb_ can undergo reduction to form a localized Mo-derived in-gap state, which traps the excess electron and limits its effectiveness as a donor. The predicted electron concentrations exceed 10^18^ cm^–3^ and 10^19^ cm^–3^ for Mo- and W-doped Sb_2_O_5_, respectively, with the electron concentration in W-doped Sb_2_O_5_ lying approximately one order of magnitude above the previously estimated Mott criterion of 1.08 × 10^18^ cm^–3^. The unfolded band structure of fully-ionized W_Sb_ shows that the W 5*d* states lie well above the conduction band edge, with minimal hybridization with the Sb 5*s*-derived CBM, thereby preserving its dispersive nature. Such weak interaction with the CBM preserves the dispersive host conduction band edge, while the moderate carrier concentrations predicted for W-doping may help avoid the stronger ionized impurity scattering associated with substantially higher doping levels. Monoclinic Sb_2_O_5_ nevertheless remains an experimentally realized but synthetically challenging TCO host, for which practical processing routes remain to be established. These findings broaden the doping mechanisms for optimizing Sb­(V)-based oxides, establishing resonant W doping as an effective route to degenerate *n*-type conductivity that is fundamentally distinct from the conventional F anion-doping mechanism reported previously.

## Supplementary Material



## Notes

Additional computational data, including competing phases convergence tests, formation energies, and chemical potential limits; extrinsic defect formation energies under metal-rich/O-poor conditions; transition level diagrams and corresponding defect/carrier concentration plots under experimentally derived chemical potential limits; projected density of states comparisons for F_O_, W_Sb_, and Mo_Sb_ for W- and Mo-doped Sb_2_O_5_ (PDF)
