# Supplementary material for: Resonant Doping in Binary Sb(V)-Oxide Sb2O5 for High-Mobility Transparent Conductors
Source: Chem Mater. 2026 Sep 11;38(18):9253–60. doi: 10.1021/acs.chemmater.6c01056 (PMC13602852; doi:10.1021/acs.chemmater.6c01056)
Supplement: Supplementary file 1 [file cm6c01056_si_001.pdf]

Resonant Doping in Binary Sb(V)-oxide Sb_2O_5 for High-Mobility Transparent Conductors

Ke Li,[†] Romain Claes,[‡] Alexander G. Squires,[‡] and David O. Scanlon[*,‡]

†*Department of Chemistry, University College London, 20 Gordon Street, London WC1H 0AJ, UK*

‡*School of Chemistry, University of Birmingham, Edgbaston, Birmingham, B15 2TT, UK*

E-mail: d.o.scanlon@bham.ac.uk

Phone: +44 (0)20 7679 4558

Supplementary Information contains convergence and formation energies for the competing phases, and chemical potential limits for W- and Mo-doped Sb_2O_5 under both *p*- and *n*-type conditions. All extrinsic defect formation energy tables under *n*-type conditions for both systems at their corresponding self-consistent Fermi levels are also included. A comparison of the projected density of states of F_O, W_{Sb}, and Mo_{Sb} is provided.

Doping Behavior of W and Mo in Sb_2O_5

Table 1: Formation energies per formula unit (ΔE_f) of W-doped Sb_2O_5 and all competing phases, with k-meshes used in calculations. Only the lowest energy polymorphs are included.

Formula	Space Group	k-mesh	ΔE_f (eV/fu)
Sb_2O_5	C2/c	6×6×5	-9.321
W	Im$\overline{3}$m	16×16×16	0.000
Sb	R$\overline{3}$m	11×11×11	0.000
O_2	P4/mmm	1×1×1	0.000
WO_3	P$\overline{4}2_1$m	3×3×6	-8.559
SbO_2	Pna2_1	4×4×2	-4.282

Table 2: Chemical potential limits for W-doped Sb_2O_5 under *p*- and *n*-type conditions.

Limit	Sb	O	W	W-Limiting Phase
Sb_2O_5-O_2 (O-rich/metal-poor)	-4.66	0.00	-8.56	WO_3
Sb_2O_5-SbO_2 (metal-rich/O-poor)	-2.77	-0.76	-6.29	WO_3

Table 3: The DOPED-generated extrinsic defect formation energy table for W-doped Sb_2O_5 under the most *n*-type conditions, showing all the energy contributions in Equation 1 used to compute the defect formation energy ($\Delta E_f^{D,q}$) All formation energies are calculated at the predicted self-consistent Fermi level of $E_F = 3.24$ eV. [1,2]

Defect	q	ΔE^{raw}	$q\epsilon_{vbm}$	qE_F	$\sum_i n_i E_i$	$\sum_i n_i \mu_i$	E^{corr}	$\Delta E_f^{D,q}$
${W_{Sb}}^{+1}$	+1	-20.63	4.00	3.24	10.58	3.52	0.08	0.79
${W_{Sb}}^{0}$	0	-12.92	0.00	0.00	10.58	3.52	0.00	1.17
${W_{i(C1,O1.97)}}^{+6}$	+6	-59.45	24.00	19.44	16.20	6.29	5.74	12.22
${W_{i(C1,O1.97)}}^{+5}$	+5	-52.54	20.00	16.20	16.20	6.29	4.12	10.27
${W_{i(C1,O1.97)}}^{+4}$	+4	-44.76	16.00	12.96	16.20	6.29	2.95	9.64
${W_{i(C1,O1.97)}}^{+3}$	+3	-37.24	12.00	9.72	16.20	6.29	1.87	8.84
${W_{i(C1,O1.97)}}^{+2}$	+2	-26.61	8.00	6.48	16.20	6.29	0.82	11.17
${W_{i(C1,O1.97)}}^{+1}$	+1	-22.02	4.00	3.24	16.20	6.29	0.46	8.16
${W_{i(C1,O1.97)}}^{0}$	0	-13.59	0.00	0.00	16.20	6.29	0.00	8.89

Table 4: Formation energies per formula unit (ΔE_f) of Mo-doped Sb_2O_5 and all competing phases, with k-meshes used in calculations. Only the lowest energy polymorphs are included.

Formula	Space Group	k-mesh	ΔE_f (eV/fu)
Sb_2O_5	C2/c	6×6×5	-9.321
Mo	Im$\bar{3}$m	7×17×17	0.000
Sb	R$\bar{3}$m	11×11×11	0.000
O_2	P4/mmm	1×1×1	0.000
MoO_3	Pc	4×4×3	-7.780
SbO_2	Pna2_1	4×4×2	-4.282

Table 5: Chemical potential limits for Mo-doped Sb_2O_5 under p- and n-type conditions.

Limit	Sb	O	Mo	Mo-Limiting Phase
Sb_2O_5-O_2 (O-rich/metal-poor)	-4.66	0.00	-7.78	MoO_3
Sb_2O_5-SbO_2 (metal-rich/O-poor)	-2.77	-0.76	-5.51	MoO_3

Table 6: The DOPED-generated extrinsic defect formation energy table for Mo-doped Sb_2O_5 under the most n-type conditions, showing all the energy contributions in Equation 1 used to compute the defect formation energy ($\Delta E_f^{D,q}$) All formation energies are calculated at the predicted self-consistent Fermi level of $E_F = 3.18$ eV. [1,2]

Defect	q	ΔE^{raw}	$q\epsilon_{vbm}$	qE_F	$\sum_i n_i E_i$	$\sum_i n_i \mu_i$	E^{corr}	$\Delta E_f^{D,q}$
${Mo_{Sb}}^{+1}$	+1	-17.66	4.00	3.18	8.42	2.74	0.09	0.77
${Mo_{Sb}}^{0}$	0	-10.40	0.00	0.00	8.42	2.74	0.00	0.75
${Mo_{i(C1,O1.97)}}^{+6}$	+6	-56.68	24.00	19.08	14.04	5.51	5.80	11.75
${Mo_{i(C1,O1.97)}}^{+5}$	+5	-50.50	20.00	15.90	14.04	5.51	4.17	9.11
${Mo_{i(C1,O1.97)}}^{+4}$	+4	-43.38	16.00	12.72	14.04	5.51	2.85	7.74
${Mo_{i(C1,O1.97)}}^{+3}$	+3	-35.90	12.00	9.54	14.04	5.51	1.88	7.07
${Mo_{i(C1,O1.97)}}^{+2}$	+2	-27.76	8.00	6.36	14.04	5.51	1.11	7.26
${Mo_{i(C1,O1.97)}}^{+1}$	+1	-19.91	4.00	3.18	14.04	5.51	0.44	7.26
${Mo_{i(C1,O1.97)}}^{0}$	0	-12.23	0.00	0.00	14.04	5.51	0.00	7.32

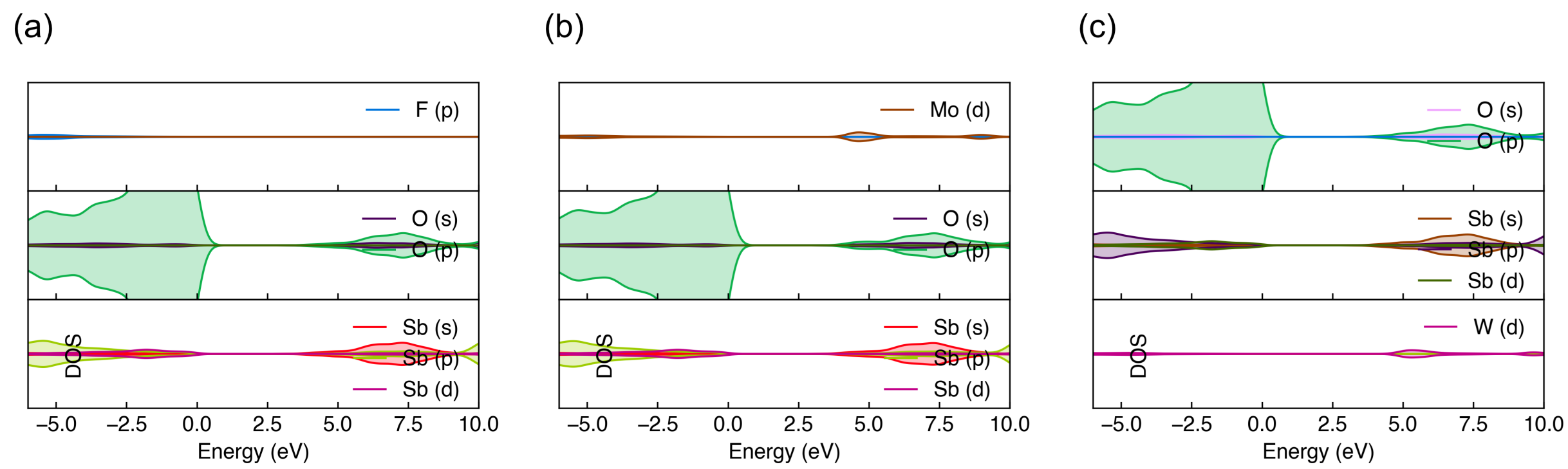

Figure 1: Element-projected density of states for (a) ${F_O}^{+1}$, (b) ${Mo_{Sb}}^{+1}$, and ${W_{Sb}}^{+1}$ in Sb_2O_5. Contributions from O, Sb and dopant orbitals are shown separately.

References

(1) Kavanagh, S. R.; Squires, A. G.; Nicolson, A.; Mosquera-Lois, I.; Ganose, A. M.; Zhu, B.; Brlec, K.; Walsh, A.; Scanlon, D. O. Doped: Python Toolkit for Robust and Repeatable Charged defect Supercell Calculations. *JOSS* **2024**, *9*, 6433.

(2) Squires, A. G.; Scanlon, D. O.; Morgan, B. J. Py-Sc-Fermi: Self-Consistent Fermi Energies and Defectcon centrations from Electronic Structure Calculations. *JOSS* **2023**, *8*, 4962.
